# Supplementary material for: Characterization of the Water Shortage Effects on Potato Tuber Tissues during Growth Using MRI Relaxometry and Biochemical Parameters
Source: Plants (Basel). 2022 Jul 25;11(15):1918. doi: 10.3390/plants11151918 (PMC9330452; doi:10.3390/plants11151918)
Supplement: Supplementary file 1 [file plants-11-01918-s001.zip › plants-1792355-supplementary.pdf]

Supplementary Materials

Supplementary Figure S1

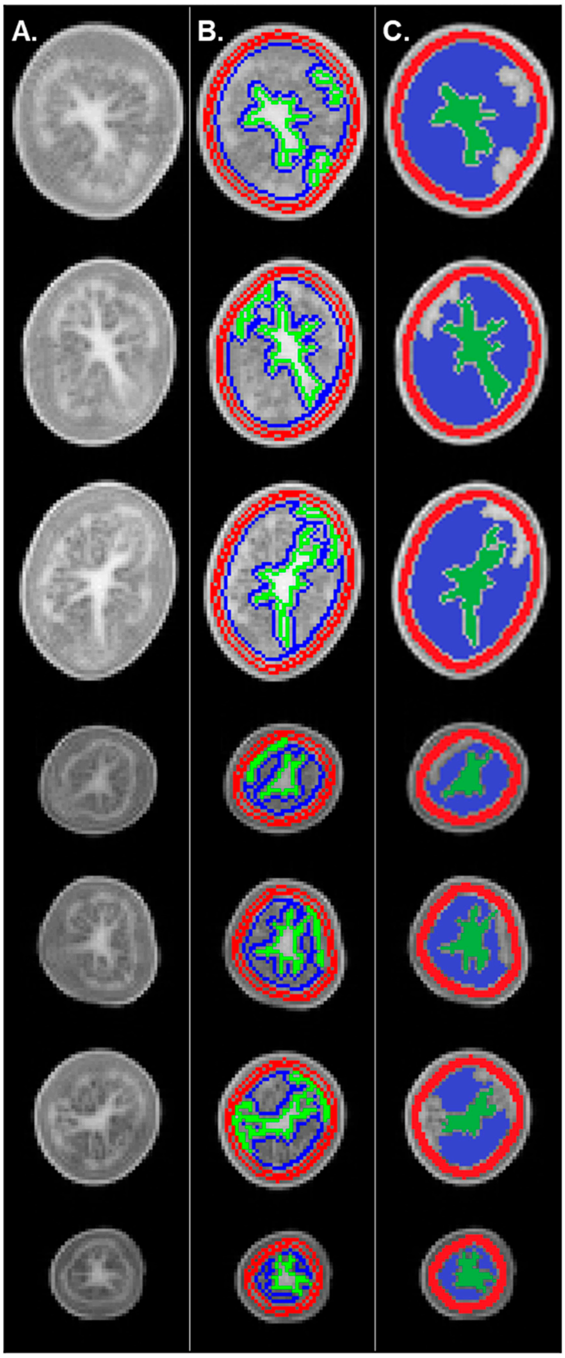

Supplementary Figure S1. A. Multi-spin-echo MRI images (echo time = 130 ms, repetition time = 10 s, 0.8 × 0.8 × 5 mm^3 voxel) of potato tubers at 60 days after shoot emergence (DASE). **B.** Masks of the three different tissues (cortex, pith, flesh) obtained by means of an automatic segmentation algorithm developed for transverse relaxation time (T_2) computation. **C.** The three different regions obtained following segmentation according to T_2: cortex (red), flesh (blue) and pith (green).

Supplementary Figure S2

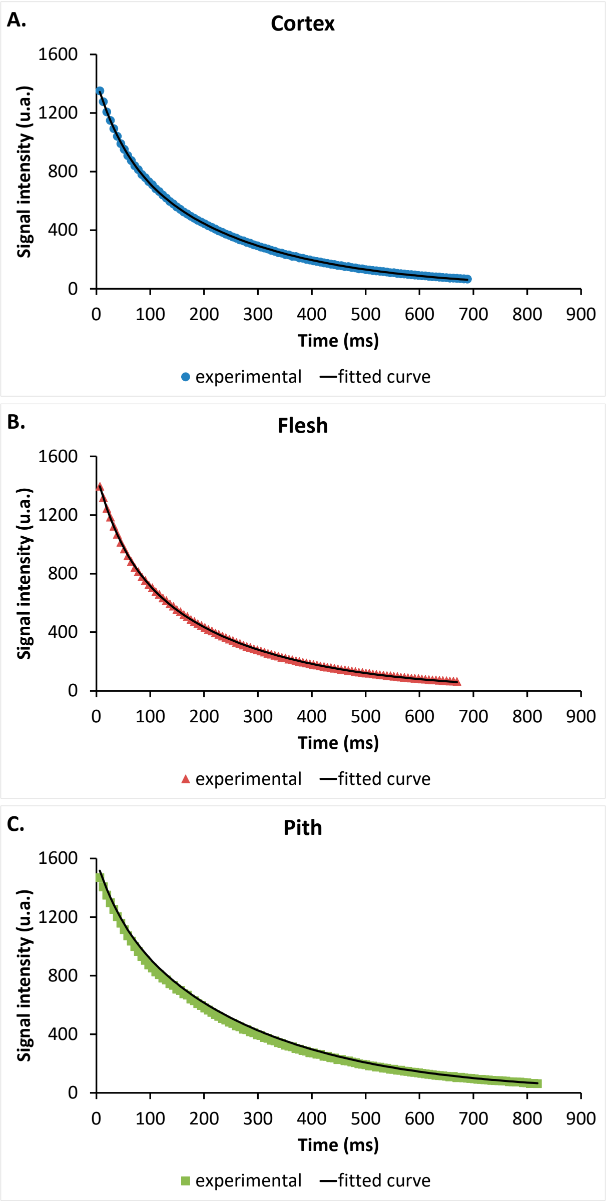

Supplementary Figure S2. T_2 decay curves generated from the computation of the mean signal in selected tuber tissue regions of interest (**A.** Cortex, **B.** Flesh, **C.** Pith) from all Mutli-Spin Echo sequence images. Values corresponding to a signal-to-noise ratio lower than 7 were removed from the T_2 curves before fitting according to the Levenberg-Marquardt algorithm using TableCurve software. Optimal fitting was reached using a bi-exponential model.

Supplementary Figure S3

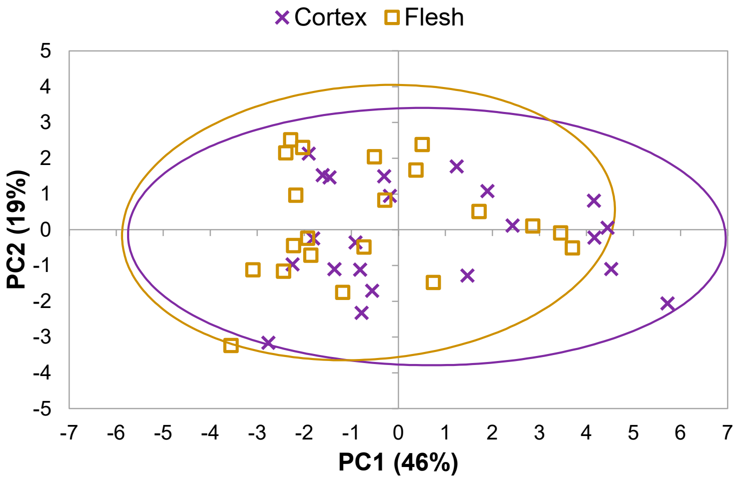

Supplementary Figure S3. Principal component analysis (PCA) score plot derived from data obtained from MRI relaxometry and physiological measurements of potato tuber tissues using principal components 1 (PC1) and 2 (PC2). Sample scores are differentiated by colour and symbol to indicate sample classification according to tissue type. Continuous lines shown on the score plot represent the ellipsis of confidence at 95% for each group.

Supplementary Figure S4

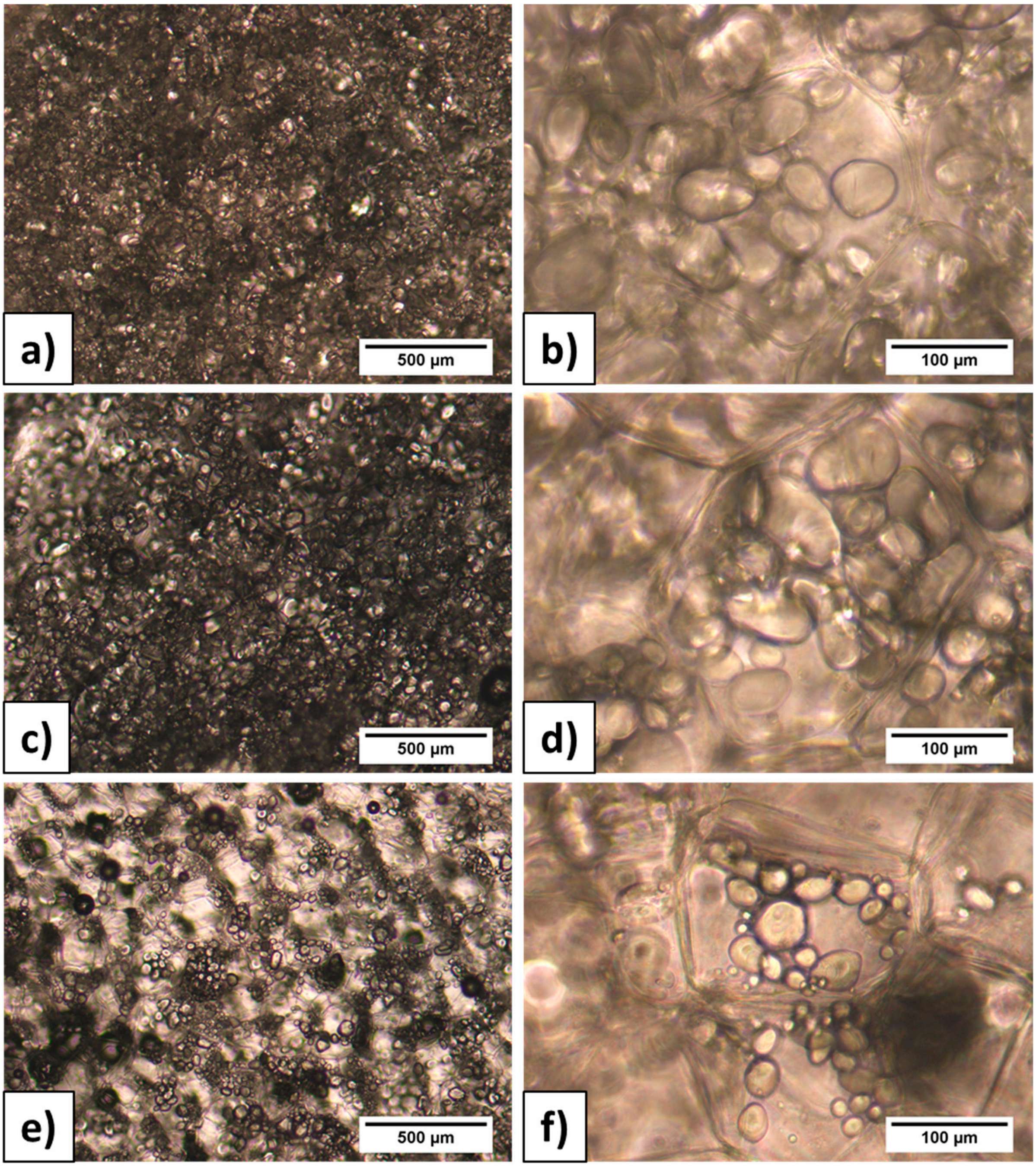

Supplementary Figure S4: Optical micrographs obtained using a Nikon Eclipse 80i of a 1 mm-thick slice of potato tissue of the cortex (**a** and **b**), the perimedullary region (**c** and **d**) and the pith (e and f) of the potato tuber. The tissue was washed with water to remove all excess starch.

Supplementary Figure S5

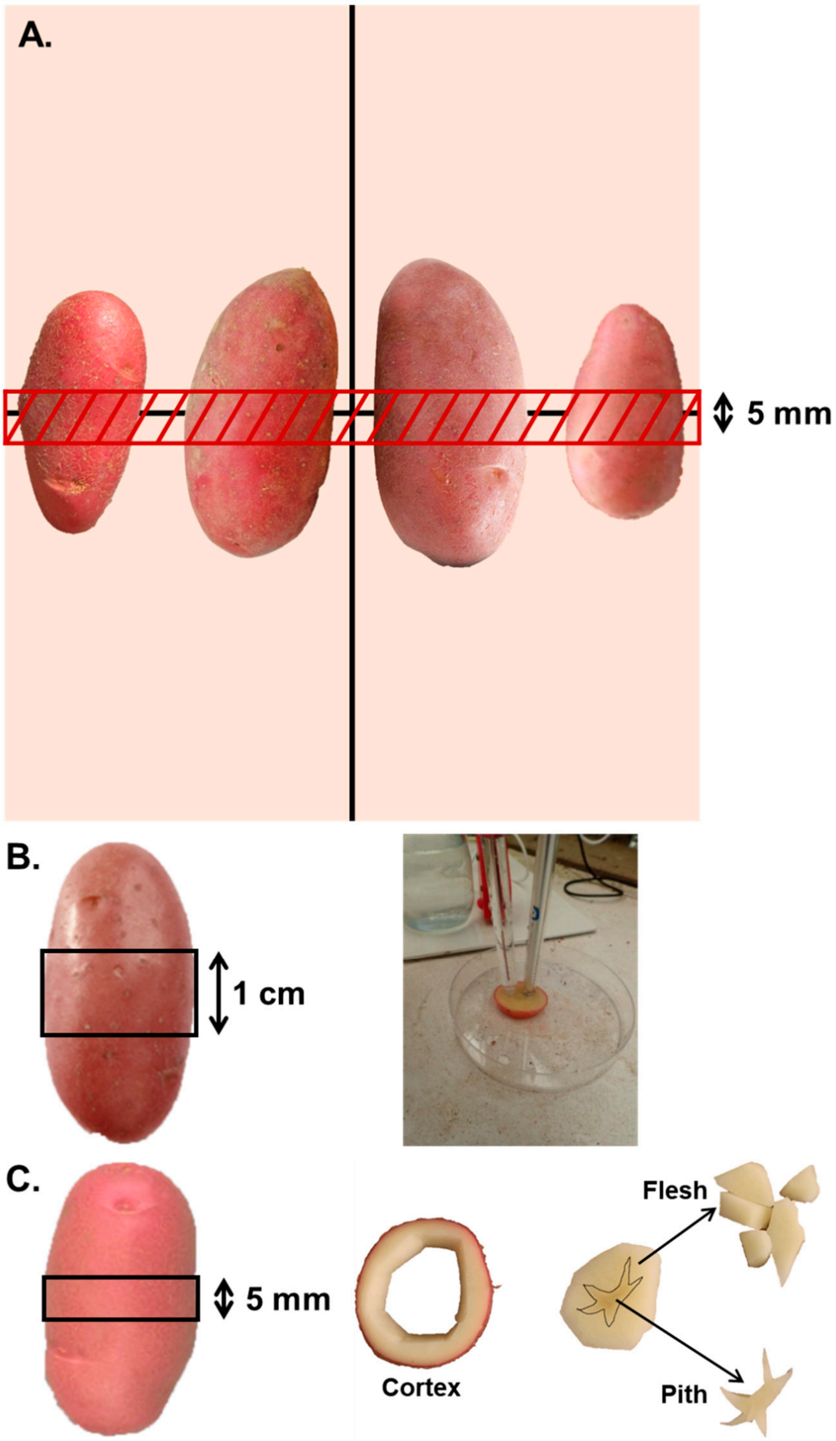

Supplementary Figure S5. A. Arrangement of potato tubers on a rectangular tray designed for insertion in a box placed in the temperature-regulating device installed inside an RF coil for transverse relaxation time measurements by MRI. **B.** Redox-potential measurement of tissue from the cross section of a potato tuber. **C.** Separation of tissues in a potato slice for metabolite analysis.
